# Effect of resistance exercise on attentional bias toward food in overweight female college students: An event-related potential study

**DOI:** 10.1016/j.ijchp.2025.100632

**Published:** 2025-10-06

**Authors:** Jifu Wang, Feng Ding, Shuailei Lian, Lin Yu

**Affiliations:** a College of Education and Sports Sciences, Yangtze University, Jingzhou, China; b Neurocognition and Action - Biomechanics Research Group, Faculty of Psychology and Sports Science, Bielefeld University, Bielefeld, Germany; c Center for Cognitive Interaction Technology (CITEC), Bielefeld University, Bielefeld, Germany

**Keywords:** Resistance exercise, Attentional engagement/disengagement, Food cues, Overweight female undergraduates, Event-Related Potentials (ERP)

## Abstract

**Background:**

Attentional bias toward high-energy foods may increase appetite, leading to overconsumption and overweight. Physical exercise has been shown to reduce such bias, however, limited research has investigated its effects and underlying mechanisms.

The present study aimed to explore whether acute resistance exercise modulates attentional bias toward food-related stimuli among young overweight women.

**Methods:**

Forty-three overweight female college students were randomly assigned to either an experimental group (*n* = 20; BMI = 25.25 ± 0.84) that performed 41 min of moderate-intensity resistance exercise or a control group (*n* = 23; BMI = 25.52 ± 1.01) that completed a reading task. Attentional bias was assessed using a dot-probe task with high- and low-energy food images after the exercise or control session, with the behavioral (reaction time, accuracy, attentional engagement/disengagement index) and neurophysiological (N2, P3) measures.

**Results:**

Compared to the control group, the exercise group had a significantly lower attentional engagement index for the low-energy food cues and significantly shorter peak latency of N2 and P3 during the dot probe task. Within the experimental group, the N2 peak amplitude was significantly lower in the high-energy vs. low-energy condition when there were incongruent food cues.

**Conclusion:**

These results indicate that the onset of attentional engagement and attentional orientation toward food cues occurred significantly earlier after resistance exercise. This study provides novel insights into the neurocognitive mechanisms underlying resistance exercise-induced modulations of attentional processing of food-related stimuli in overweight females, offering both theoretical contributions to exercise cognition and practical implications for weight management interventions.

## Introduction

The prevalence of overweight and obesity has been increasing at a worrying rate in China. In 2018, the Chinese Centers for Disease Control and Prevention found that obesity (body mass index, BMI ≥ 28kg/m^2^) in adults was 8.1 %. The prevalence of obesity in adults increased to 14.1 % in 2023 (Beals et al., 2023). Being overweight or obese could significantly increase the probability of having chronic diseases such as hypertension and diabetes (Mutsert et al., 2014). Therefore, there is a need to develop effective methods to prevent weight gain. The present study focused on biased attention to food cues as a potential contributor to overweight. We aimed to determine whether resistance exercise could reduce the biased attention of overweight individuals toward such cues.

Attentional bias refers to the selective allocation of attention resources to certain stimuli (Roy et al., 2008). Individuals tend to show attentional bias to food-related stimuli (van Ens et al., 2019). However, high attentional bias to food cues seems to be a potential contributor to overeating (Kakoschke et al., 2015). Attentional bias to food information can increase appetite, which may lead to excessive food intake and health problems such as overweight or obesity (Zhang et al., 2018). The cognitive motivation model (Veenstra-Wijnen, 2011) suggests that people who are overweight are more sensitive to high-energy foods. Congruent with this model, compared with people who were of normal weight, people who were overweight devoted more attention resources to high-energy foods, and reward areas in the cortex were more highly activated when high-energy food information was presented (Veenstra-Wijnen, 2011). Overweight individuals exhibited difficulties in disengaging from high-calorie, appetizing food stimuli, as demonstrated through the dot probe paradigm (Castellanos et al., 2009), which seems to be one of the most widely used paradigms for attentional bias studies.

Several studies have demonstrated that engaging in physical exercise can reduce attentional bias toward food-related stimuli. The attentional bias was significantly increased among overweight and obese individuals after expending 500 kcal through aerobic exercise (Flack et al., 2022). Moderate- or high-intensity resistance exercise has also been shown to improve the attentional engagement of heroin-dependent individuals (Tao et al., 2020). A 34-minute high-intensity interval exercise has been demonstrated to effectively enhance individuals’ focus on health-related attributes when making food choices (Liu et al., 2021). Exercise-induced improvements in attention and processing speed in patients with ADHD demonstrated that adult patients with ADHD may benefit from an acute bout of exercise (Mehren et al., 2019). Another study revealed that acute exercise exerted a more pronounced influence on picture-based attentional bias than on word-based attentional bias (Cooper & Tomporowski, 2017). In terms of exercise types, high-intensity interval training (HIIT, Liu, 2021), brisk walking (Oh & Taylor, 2013), tai chi (Cui, 2020), and other exercises have been shown to reduce attention bias toward high-energy foods. Moreover, Comstock et al. (2013) found that acute resistance exercise was beneficial in maintaining lean body mass and reducing fat mass. Therefore, resistance exercise was chosen as the intervention method for the study.

To date, research investigating the impact of exercise on attentional bias toward food has primarily relied on behavioral measures, such as reaction time and accuracy of the dot probe task. Little research has employed electroencephalogram (EEG) or event-related potentials (ERPs) as a measure of attentional processes in such context. In the present study, we investigated whether resistance exercise could reduce attention bias toward food, utilizing ERPs to uncover the underlying neurophysiological mechanisms associated with this effect. ERPs provide a fine-grained perspective on the temporal dynamics of neural processes involved in attentional processing, which could offer deeper insights beyond behavioral measurements. N2 and P3 components are widely utilized as indicators of neural activity in the early stages of attentional processing. N2 is a negative component that peaks around 200 ms after the stimulus onset. A larger amplitude of N2 has been associated with the investment of cognitive resources in an attention task (Huster et al., 2013). Moreover, the high-energy food pictures have elicited a shorter N2 latency and a larger N2 amplitude than the low-energy food pictures (Kong et al., 2011). P3 is a positive component that peaks around 300 ms after the stimulus onset. The amplitude of the P3 component is believed to reflect the distribution of intentional attentional resources (Schupp et al., 2006). Larger P3 amplitudes were observed toward food stimuli than neutral stimuli in both normal and overweight/obese participants (Nijs et al., 2010). Moreover, research also revealed that, compared to participants with normal weight, overweight individuals exhibited shorter P3 latency during a task designed to measure attentional bias toward images of appetizing foods (Hume et al., 2015). Furthermore, according to a recent study (Xie et al., 2024), participants exhibited increased P3 amplitudes toward high-energy foods following acute HIIT relative to the control session.

Previous studies have documented that overweight individuals show attentional bias toward food-related cues (i.e. high-energy foods), and physical exercise could reduce such attentional bias. However, the neurocognitive mechanisms underlying the reduction of such attentional bias remain poorly understood. Few studies have investigated the relationship between resistance exercise and neurophysiological measures of neural activity underlying food attentional bias. The aim of the current study is to explore the efficacy of acute resistance exercise in modulating attentional bias toward food-related stimuli among overweight females. Compared to males, female individuals reported higher levels of concern about their weights (Grabe et al., 2008 for a meta-analysis; Neighbors et al., 2007), engaged in more dieting behaviors (Striegel-Moore et al., 2009), and were diagnosed with eating disorders at significantly higher rates (Capuano et al., 2025 for a review). Therefore, we selected overweight young women as the participants and explored the resistance exercise effects on their food-related attention. Participants were randomly assigned to either the experimental group (resistance exercise) or the control group (reading task). The dot detection paradigm was used to test the effect of the resistance exercise on attention bias toward different food cues, and ERP data were used to identify a mechanism of this effect. Based on the cognitive motivation model (Veenstra-wijnen, 2011), we hypothesized that (a) attention bias toward high-energy foods will be lower in the resistance exercise vs. control group, (b) there will be shorter ERP latency and larger amplitude at N2 and P3 in the resistance exercise vs. control group, and (c) participants in both groups will show longer RT and a lower index of attentional engagement in response to high-energy vs. low-energy foods.

## Methods

### Participants

G*Power 3.1.9 was used for a priori power analysis (Faul et al., 2007). The calculation was based on a 2 (Group: resistance exercise vs. control; between-subjects factor) × 2 (Picture Type: high-energy vs. low-energy; repeated-measures factor) × 2 (Cue congruence: congruent vs. incongruent; repeated-measures factor) mixed-design analysis of variance (ANOVA). The sample size needed to detect a medium effect size (*f* = 0.25) with power = 0.80 was 24.

To increase the robustness and reproducibility of the experimental results, we recruited 43 participants in the study. All of them were female students in college who were overweight, with a BMI between 24 and 28 (according to the Chinese National Standard of Student Physical Health). Participants were randomly assigned to either the experimental group or the control group, with 20 individuals in the experimental group and 23 in the control group. The mean age of the participants was 21.25 (*SD* = 1.48). All participants were right-handed and had normal or corrected-to-normal vision. Moreover, the inclusion criteria of participants were as follows, all based on their self-report: (1) no eating or psychological disorders; (2) no congenital obesity, diabetes, or cardiovascular or nervous system diseases; (3) non-vegetarian; (4) no weight loss medication taken in the past month; (5) no food-intake during the two hours before the experiment. To avoid differences caused by diet, the experiment was conducted from 19:00 to 21:00. All participants gave their written informed consent following the Declaration of Helsinki. The protocol of the current experiment was approved by the Human Experiment Ethics Committee of the first author’s affiliated university (Project identification code: 2022,018).

### Design and task

We employed a 2 (*group*: resistance exercise vs. control) × 2 (*picture type*: high-energy vs. low-energy) × 2 (*cue congruence*: congruent vs. incongruent) mixed experimental design. The *group* was a between-subject factor. The *picture type* and *cue congruence* were within-subject factors. The dependent variables were reaction time (RT) and accuracy on the attention bias task, as well as the peak amplitude and peak latency of N2 and P3 time-locked to the stimuli (pictures).

The dot probe task (Broadley et al., 2019; Stojek et al., 2018 for a review) has been used to measure the attentional bias toward food. Apart from the classical parameters RT and accuracy, the attentional engagement index and difficulty in disengagement index (DID index) were also used by researchers to evaluate attentional bias in dot probe paradigm (Franja et al., 2021; Koster et al., 2004; MacLeod et al., 1986). The attentional engagement index was calculated as the RT difference between the neutral-neutral pairs and food-neutral pairs under the congruent condition (*RT_congruent “neutral-neutral”_* – *RT_congruent “food-neutral”_*, Franja et al., 2021; Jin et al., 2023; Veenstra et al., 2012). Positive values indicate attentional bias toward food related neutral stimuli (increased attentional engagement with the food cue), and negative values indicate attentional bias toward neutral stimuli related food (decreased attentional engagement with the food cue). The DID index was calculated as the RT difference between for food-neutral pairs and the under the neutral-neutral pairs under the incongruent condition (*RT_incongruent “food-neutral”_* – *RT_incongruent “neutral -neutral”_*, El Khoury-Malhame et al., 2011; Koester et al., 2004; Jin et al., 2023). A positive DID value indicates difficulty in disengaging attention from food pictures, whereas a negative DID value indicates attentional avoidance of food pictures.

There were three types of food cues in the attention bias (dot probe) task: (a) a high-energy food picture was presented with a neutral picture; (b) a low-energy food picture was presented with a neutral picture; and (c) a neutral picture was presented with another neutral picture. In the congruent condition, the probe dot appeared on the side of the high-energy food picture rather than on the side of the neutral picture, or it appeared on the side of the low-energy food picture rather than on the side of the neutral picture. In the incongruent condition, the probe dot appeared on the side of the neutral picture.

## Materials

### Self-reported eating characteristics

To avoid the effect of eating habits, food preferences, and personality traits on the experimental results, the Dutch Eating Behavior Questionnaire (DEBQ) (Van Strien et al., 1986) was used to evaluate the participants’ eating characteristics. This questionnaire consists of 33 items on three dimensions: restrictive diet, emotional diet, and extroverted diet. Each item was rated on a 5-point Likert scale. The total score on the DEBQ has been shown to be correlated with the severity of dietary problems (Van strien et al., 1986). In the current study, Cronbach’s α was 0.88 for the full-scale, indicating good internal congruence.

### Food and neutral stimuli

Following a previous study (Huang et al., 2020), the initial set of food pictures included 60 high-energy food pictures (such as cakes, steak, and fried chicken), 60 low-energy food pictures (such as vegetables and fruits), and 30 neutral pictures (such as pens, scissors, and tables). Adobe Photoshop was used to edit the images, and the size of the pictures was 6 × 8 cm. A group of 40 undergraduate students, who were not part of the main experiment, evaluated each image based on its pleasure and arousal levels (five-point Likert). Additionally, for the food-related pictures, they also assessed how delicious the items appeared. According to the ratings, 30 high-energy food pictures, 30 low-energy food pictures, and 30 neutral pictures were finally selected. The ratings of pleasure and arousal were significantly higher for the high-energy food pictures than the low-energy food pictures (*p* < .01) and were significantly higher for the low-energy food pictures than the neutral pictures (*p* < .01). The deliciousness ratings were higher for the high-energy food pictures than for the low-energy food pictures (*p* < .01).

### Intervention protocol

Participants in the exercise group completed a 41-minute resistance exercise session (5-minute warmup, 31 min of exercise, and 5-minute recovery time). They wore a Polar bracelet to record heart rates in real time. The exercise protocol was designed according to a previous study (Li et al., 2021). During the 5-minute warmup, the participants did a set of radio gymnastics. Then they used 5-kilogram barbells for four actions: standing barbell bending, standing barbell pushing, barbell squatting, and barbell lunging. The above-mentioned four actions were carried out in sequence, which formed an action set. Within each set, the first three actions were each followed by a thirty-second rest; after the fourth action, there was a three-minute rest before the next set. These sets were repeated four times for a total exercise time of 31 min. The participants then did stretches during the 5-minute recovery time. The average heart rate was 126.45 ± 16.82 times/min, and the average sport intensity was 66.15 % ± 8.09 % HRmax. Thus, the resistance exercise reached moderate intensity. Participants in the control condition completed a 41-minute reading task in which they were asked to read the psychological literatures (i.e., *Intimate Relationships* by Rowland Miller & Daniel Perlman; *Maybe You Should Talk to Someone* by Lori Gottlieb) in a quiet room.

## Procedure

### Preparation

After the participants had finished the consent form and the DEBQ, the experimenter introduced the rules of the resistance exercise, and the participants carried out the exercises. Right after the exercises, they were then comfortably seated at the experimental desk with a monitor positioned approximately 70 cm in front of them and prepared with the EEG cap. Participants were instructed to sit upright and not to move their heads/legs during the experiment.

### Practice and formal experiment

**Fig. 1:**
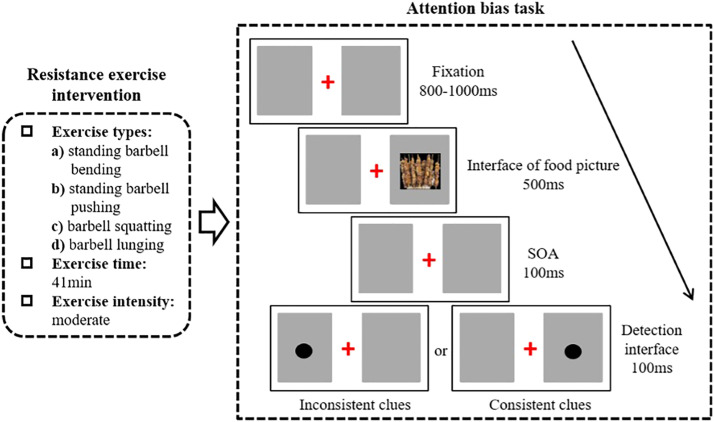
Flow chart of one experimental trial.

Before the formal experimental trials, participants performed 12 practice trials to familiarize themselves with the task. Feedback (correct or incorrect) was provided in these practice trials (but not in the formal experiment). There were 400 trials in the formal experiment, with 80 trials for high-energy food pictures and congruent cues, 80 trials for high-energy food pictures and incongruent cues, 80 trials for low-energy food pictures and congruent cues, 80 trials for low-energy food pictures and incongruent cues, and 80 trials for neutral picture pairs. The process is shown in Fig. 1. The center of the food picture was 4.7° from the center of the screen. The size of the dot was 0.3° When the dot appeared on the left side, the participants needed to press the button “s” on the keyboard, while when the dot appeared on the right side, the participants were instructed to press “k” on the keyboard.

### Data recording and analysis

#### Behavioral data

The experiment was programmed with the E-Prime 2.0 software (Psychology Software Tools, Pittsburgh, PA), and participants’ RT, as well as the accuracy of the response, were automatically recorded. The IBM SPSS 17.0 software was used to preprocess and analyze the RT, accuracy, attentional engagement index, and DID index. Trials with extreme RTs (<200 ms, or outside of mean ± three standard errors) were excluded. A mixed repeated measure analysis of variance (ANOVA) with the factors *group* (resistance exercise vs. control), *picture type* (high-energy vs. low-energy), and *cue congruence* (congruent vs. incongruent) was conducted for each dependent variable separately (RT, accuracy, attentional engagement index, and DID index).

#### Neurophysiological data

EEG signals were recorded by a 64-channel amplifier with the Brain Vision Recorder software (Brain Products, Munich, Germany). Recordings were made from 64 gel-based Ag/AgCl electrodes, which were positioned in accordance with the international 10–10 system. The electrode AFz was selected as the recording ground, and the electrode FCz was selected as the online reference. The impedance of all electrode sites was under ten kΩ. All signals were sampled at 1000 Hz before digitization and filtered from 0.01 to 100 Hz during the recording.

EEG signals were processed offline with the Brain Vision Analyzer 2.0 software. An Infinite impulse response (IIR) filter was applied to the signals with a range from 0.01 to 35 Hz. Then the signals were re-referenced to the bilateral mastoid electrodes (TP9 and TP10). With the help of independent component analysis (ICA), semi-automatic progress was used on the continuous signals to eliminate the ocular components in the blink interval. The length and bad interval free for the ICA interval were both 50 s. Then a 1200 ms epoch (time-locked to the onset of the food picture) was extracted from the continuous signals, and baseline correction was made with the first 200 ms of the epoch. The average number of artifact-free trials included in the ERP averages was approximately 70 across all experimental conditions. No significant differences in trial counts were observed between *group, picture type*, or *cue congruence* (all *p*s > 0.05).

The electrode sites and time windows were selected based on the grand average of ERPs and the results of previous studies (Stroop et al., 1992; Kong et al., 2011). The sites FCz, Cz, CPz, Oz, and POz (150–300 ms) were selected for the N2 peak amplitude and latency. The sites FCz, Cz, CPz, Pz, and Fz (250–450 ms) were selected for the P3 peak amplitude and latency. The peak amplitude and latency were measured by the baseline peak within the time window. A mixed repeated measure ANOVA was conducted, with the factors *group* as the between-subjects variable; *cue congruence, picture type*, and *electrode site* as repeated measure factors for each of four dependent measures, namely N2 peak latency, N2 peak amplitude, P3 peak latency, and P3 peak amplitude.

For the above-mentioned ANOVAs, the Greenhouse-Geisser correction was applied whenever the sphericity assumption was violated. Partial eta-squared ($\eta_{p}^{²}$) was used to evaluate the effect size. Post hoc multiple comparisons among means were made with Tukey's Honestly Significant Difference (HSD) tests.

## Results

### Homogeneity test

The homogeneity of participants' demographic and anthropometric characteristics, including age, height, weight, BMI, and eating behavior patterns, was statistically evaluated between the groups. These variables were homogeneous in both groups before the experiment, and no group differences were found on any of these variables (as shown in Table 1). Besides, the results of DEBQ showed that 43 participants had no eating behavior problems.

**Table 1:** Differences in demographic characteristics and dietary behaviors among different groups before intervention.

		Exercise (M ±SD)	Control (M±SD)	*t*	*p*
Demographic variables	Age (years)	21.25 ± 1.48	20.87 ± 1.87	0.732	.468
	Height (m)	1.62 ± 0.05	1.61 ± 0.04	0.225	.823
	Weight (kg)	66.04 ± 3.85	66.58 ± 5.33	−0.38	.706
	BMI	25.25 ± 0.84	25.52 ± 1.01	−0.959	.343
Dietary behavior	Restricted eating	3.35 ± 0.66	2.94 ± 0.83	1.76	0.18
	Emotional eating	2.50 ± 0.88	2.56 ± 0.86	−0.25	0.81
	External eating	3.36 ± 0.37	3.17 ± 0.44	1.54	0.13

Notes: The average value of DEBQ ranges from 1 to 5. A higher score indicates a more severe eating behavior.

### Behavioral data

For RT, no significant main effect of *group* was found, *F*(1, 41) = 0.176, *p* > .05, $\eta_{p}^{²}$= 0.07. The main effect of *cue congruence* was not significant either, *F*(1, 41) = 3.11, *p* > .05, $\eta_{p}^{²}$= 0.07. The main effect of *picture type* was significant, *F*(1, 41) = 10.14, *p* < .01, $\eta_{p}^{²}$= 0.20, as shown in Table 2. Post hoc testing showed that the RT was significantly longer for high-energy food pictures than for low-energy food pictures, regardless of whether the cue was congruent or incongruent. There were no significant interaction effects. No significant main effects or interaction effects were found for accuracy (all *p*s > 0.05).

**Table 2:** RT and accuracy on the dot detection task measure of food attention bias for two calorie levels and two cue congruence in the exercise and control groups (M±SD).

Dependent variable	Group	High calorie		Low calorie	
		Congruent cues	Incongruent cues	Congruent cues	Incongruent cues
RT (ms)	Exercise	407.11 ± 40.52	418.71 ± 42.75	405.82 ± 39.18	417.93 ± 46.02
	Control	419.19 ± 60.69	423.36 ± 65.41	411.13 ± 60.70	423.78 ± 73.04
Accuracy	Exercise	0.97 ± 0.24	0.97 ± 0.28	0.97 ± 0.02	0.97 ± 0.28
	Control	0.98 ± 0.02	0.97 ± 0.03	0.97 ± 0.03	0.96 ± 0.03
Attentional engagement index	Exercise	16.33 ± 28.76		16.87 ± 25.30	
	Control	18.44 ± 34.45		26.50 ± 38.43	
DID index	Exercise	−3.46 ± 19.15		−4.24 ± 26.02	
	Control	−6.90 ± 19.13		−6.48 ± 12.57	

With the attentional engagement index as the dependent variable, the main effect of *picture type* was significant, *F*(1, 41) = 4.887, *p* > .05, $\eta_{p}^{²}$= 0.107. Post hoc testing showed that the attentional engagement index was significantly larger for low-energy than high-energy food pictures. The main effect of the *group* was not significant, *F*(1, 41) = 0.363, *p* > .05, $\eta_{p}^{²}$= 0.09. There was a trend toward significance in the interaction between *picture type* and *group, F*(1, 41) = 3.743*, p* = .06$,\;\eta_{p}^{²}$= 0.084. Simple effects analysis showed that the attentional engagement index for low-energy food pictures was significantly lower (*p* < .01) in the resistance exercise group (16.87 ± 25.30) than in the control group (26.50 ± 38.43).

With the DID index as the dependent measure, the main effect of picture type was not significant, *F*(1, 41) = 0.007, *p* > .05. The main effect of group was not significant, *F*(1, 41) = 0.263, *p* > .05. The interaction between picture type and group was not significant (*p* > .05).

### Neurophysiological data

#### N2 peak latency

The main effect of the *group* was significant, *F*(1, 41) = 7.712, *p* < .01$,\;\eta_{p}^{²}$= 0.158, as shown in Fig. 1. The post hoc test showed that the N2 peak latency was significantly shorter in the resistance exercise group than in the control group. The main effect of *picture type* was not significant, *F*(1, 41) = 0.118, *p* > .05, $\eta_{p}^{²}$= 0.003. The main effect of *cue congruence* was not significant, *F*(1, 41) = 1.604, *p* > .05, $\eta_{p}^{²}$= 0.038. The main effect of the *electrode site* was significant, *F*(1.73, 70.97) = 4.199, *p* < .05$,\;\eta_{p}^{²}$= 0.093. The post hoc test showed that the N2 peak latency was longer at CPz than at POz (*p* = .09). The interaction between *electrode site* and *cue congruence* was significant, *F*(2.61, 107.08) = 4.447, *p* < .01$,\;\eta_{p}^{²}$= 0.098. Simple effects analysis showed that the N2 peak latency was significantly shorter when evoked by congruent cues than incongruent cues at Cz and FCz (*p* < .05); in the incongruent cues condition, the N2 peak latency was significantly shorter at POz than at Cz and FCz (*p* < .05). The interaction among the *group, cue congruence*, and *electrode site* was significant, *F*(4, 38) = 2.989, *p* < .05,$\;\eta_{p}^{²}$= 0.068. Simple effects analysis showed that in the control group, the N2 peak latency was significantly shorter for the congruent cues than for the incongruent cues at Cz, CPz, and FCz (*p* < .05).

#### N2 peak amplitude

The main effect of the *group* was not significant, *F*(1, 41) = 0.713, *p*>.05, $\eta_{p}^{²}$= 0.017. The main effect of *picture types* was not significant, *F*(1, 41) = 1.483, *p*>.05, $\eta_{p}^{²}$= 0.035. The main effect of *cue congruence* was not significant, *F*(1, 41) = 0.100, *p*>.05, $\eta_{p}^{²}$= 0.002. The main effect of electrode sites was significant, *F*(1.38, 56.71) = 24.237, *p* < .01, $\eta_{p}^{²}$= 0.372. Post hoc test showed that the N2 peak amplitude was significantly larger at POz than that at Oz, FCz, and Cz (*p* < .01). The interaction effect between *electrode site* and *picture type* was significant, *F*(2.40, 98.38) = 15.162, *p* < .01, $\eta_{p}^{²}$= 0.270. Simple effects analysis showed that the N2 peak amplitude was significantly larger at POz than that at Cz, CPz, and FCz regardless of the high- or low-energy food pictures (*p* < .01). The N2 peak amplitude of high-energy food pictures was significantly larger than that of low-energy food pictures at FCz (*p* < .05). The interaction effect among *picture type, cue congruence,* and *group* was significant, *F*(1, 30) = 7.396, *p* = .01, $\eta_{p}^{²}$= 0.153. Simple effects analysis showed that under the condition of incongruent cues, the N2 peak amplitude of high-energy food pictures was significantly smaller than that of low-energy food pictures in the resistance exercise group.

#### P3 peak latency

**Fig. 2:**
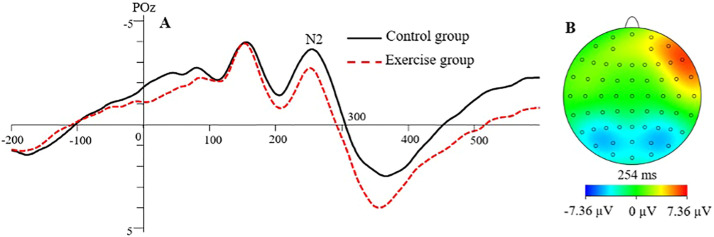
(A) The grand average waveform for N2 in the resistance exercise group and control group at the posterior electrode POz. (B) The topographic map for the differences between the resistance exercise group and the control group (exercise − control).

**Fig. 3:**
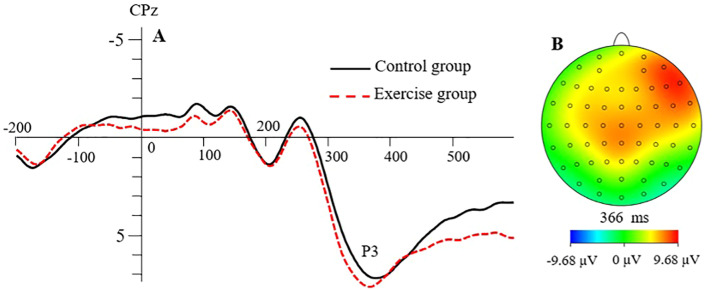
(A) The grand averaged waveform for P3 in the resistance exercise group and control group. (B) The topographic map for the differences between the resistance exercise group and the control group (exercise − control).

The main effect of the *group* was significant, *F*(1, 41) = 8.494, *p* < .01, $\eta_{p}^{²}$= 0.172, as shown in Fig. 2. The post hoc test showed that the P3 peak latency was significantly shorter in the resistance exercise group than in the control group. The main effect of *picture type* was not significant, *F*(1, 41) = 0.620, *p*>.05, $\eta_{p}^{²}$= 0.015. The main effect of *cue congruence* was not significant, *F*(1, 41) = 1.251, *p*>.05, $\eta_{p}^{²}$= 0.030. The main effect of the *electrode site* was not significant, *F*(2.35, 96.37) = 2.621, *p*>.05, $\eta_{p}^{²}$= 0.060. The interaction effects among the *group, picture type, cue congruence*, and *electrode site* were not significant (*p* > .05) (Fig. 3).

#### P3 peak amplitude

The main effect of the *group* was not significant, F(1, 41) = 1.186, *p*>.05, $\eta_{p}^{²}$= 0.028. The main effect of *picture type* was not significant, *F*(1, 41) = 2.025, *p*>.05, $\eta_{p}^{²}$= 0.047. The main effect of *cue congruence* was significant, *F*(1, 41) = 4.139, *p* < .05, $\eta_{p}^{²}$= 0.092. The post hoc test showed that the P3 peak amplitude of congruent cues was significantly larger than that of incongruent cues. The main effect of the *electrode site* was not significant, *F*(1.40, 57.18) = 2.796, *p*>.05, $\eta_{p}^{²}$= 0.064. The interaction effect for *group, picture type*, and *cue congruence* was significant, *F*(1, 41) = 7.332, *p* < .01, $\eta_{p}^{²}$=0.152. Simple effects analysis showed that under the condition of congruent cues, the P3 peak amplitude evoked by high-energy food pictures was significantly larger than that evoked by low-energy food pictures in the control group (*p* < .05). Under the resistance exercise condition, the P3 peak amplitude evoked by the low-energy food pictures was significantly larger in the congruent cues condition than that in the incongruent cues condition (*p* < .05). Under the control condition, the P3 peak amplitude evoked by high-energy food pictures was significantly larger in the congruent cues condition than that in the incongruent cues condition (*p* < .05).

## Discussion

The present study explored the efficacy of acute resistance exercise in modulating attentional bias toward food-related stimuli among young overweight women. The results showed that (1) compared to the control group, the experimental group had a significantly lower attentional engagement index for the low-energy food cues and significantly shorter peak latency of N2 and P3 during the dot detection task; (2) within the experimental group, the N2 peak amplitude was significantly lower in the high- energy vs. low-energy condition when there were incongruent food cues.

Behaviorally, compared to sedentary control group, resistance exercise group showed a significant smaller attentional engagement index to the low-energy food, which indicates that after the 41-min resistance exercise, low-energy food received less attentional engagement, in other words, less attention was paid to low-energy food cues after exercise. This finding seems to contradict our research hypothesis (a) that attentional bias toward high-energy foods will be lower in the resistance exercise than the control group. Previous studies showed that exercise may consistently reduce the liking and wanting for high-energy food (Beaulieu et al., 2020 for review), as well as the attentional bias toward high-energy, palatable food cues (Cui, 2020; Liu, 2021; Oh & Taylor, 2013). Moreover, neuroimaging studies also found that habitual physical activity and structured exercise seem to be associated with reduced brain responses to high-calorie food cues in reward regions such as insula, orbito-frontal cortex, and precuneus (Dera et al., 2023). However, we did not find the expected reduction of attentional bias towards high-energy food cues for the resistance exercise group, but a significant reduction of attentional bias towards low-energy food cues after exercise. It can be attributed by increased energy demands after exercise. After exercise, the body needs to replenish energy stores, and low-energy foods seem to be seen as less "useful" for energy refuel, which may lead to reduced attentional bias. Moreover, the reward value of high-energy foods can be enhanced after physical exercise, which makes low-energy foods less rewarding/attended in comparison (Beaulieu et al., 2020). Considering we did not find a significant increased attentional bias/engagement toward high-energy food cues after exercise (no significant difference for attentional engagement index), the exercise might “reduce” the “enhanced” attentional engagement bias towards high-energy food cues and made it no significant difference between groups.

Another explanation can be from the subjects. Overweighed participants were tested in our study, however most of the above-mentioned studies which reported the reduction of attentional bias towards high-energy food used healthy participants. Research with overweight participants (Flack et al., 2022) found the attentional bias towards high-energy food (measured by fixation durations) was significantly increased after a large dose of physical exercise (500 kcal) among the overweight and obese individuals. In a food related Flanker task, Xie et al. (2024) also found that obesity individuals responded faster for high-energy food stimuli after HIIT relative to the control session, which seems to indicate an increased attentional bias (even though the flanker task was not designed for it). High-energy foods received more attention while simultaneously reducing the attention toward low-energy foods. While, given that a limited number of literatures investigating effects of exercise on attentional bias for food cues, especially with overweight individuals, the question is still open to future studies.

Neurophysiologically, we found that the N2 evoked by the attention bias task had a shorter latency in the resistance exercise group than in the control group. The N2 component reflects both attentional orienting toward task-relevant stimulus locations (Huster et al., 2013) and strategic cognitive command of attentional control (Olson et al., 2016). The larger the N2 amplitude, the more attention resources are being invested. In our study, overweight young women accelerated attentional engagement with food-related stimuli after resistance exercise. This suggested that the desire for food increased in overweight young women after the resistance exercise. In addition, N2 elicited by the congruent cues had shorter latency than that elicited by the incongruent cues, in both exercise and control groups. The participants in both groups showed an orientation in attention to the food stimuli under the condition of incongruent cues. With such attentional orientation, longer RTs and later N2s appeared. Furthermore, when there were incongruent cues, smaller N2s were found for the high-energy food stimuli than the low-energy food stimuli in the resistance exercise group, but not in the control group. The results indicate that after acute resistance exercise, the overweight participants devoted more attention resources to low-energy food cues than high-energy food cues. These findings are congruent with other studies showing that moderate-intensity aerobic exercise could enhance appetite regulation (Martins et al., 2015) and effectively improve food decision-making (Huang et al., 2020).

In the current study, the P3 evoked by biased attention to high-energy foods had shorter latency in the resistance exercise group than in the control group. Such findings are in line with evidence that P3 latency evoked by attention bias toward delicious food pictures was shorter for obese participants than participants with normal weight (Hume et al., 2015). Given that P3 reflects the distribution of deliberate attention (Schupp et al., 2006), our result suggests that participants paid attention to the distribution of food cues earlier after acute resistance exercise. Participants in the exercise group might have felt hungrier after the exercise and, therefore, showed an increase in attention to food cues. In addition, in the resistance exercise group, larger P3s were found for the congruent cues than the incongruent cues with the low-energy food stimuli. It is suggested that the P3 amplitude may be related to the internal drive of stimulation (Feig et al., 2017). Participants in the exercise group showed a larger internal drive for low-energy foods after resistance exercise with the congruent cue. These results are congruent with other research showing that the P3 evoked by the priority attention had a larger amplitude for the food cues than that for the neutral cues (Meule et al., 2015), and that the P3 amplitude evoked by food cues was positively correlated with food craving (Iceta et al., 2020).

Taking the behavioral and the neurophysiological results together, it can be inferred that the lower attentional engagement toward low-energy food cues after the resistance exercise was related to the shorter latency of N2 and P3. Based on energy metabolism, it can be speculated that hunger levels increased significantly after resistance exercise. Participants were asked to fast for two hours prior to the experiment, therefore, the attentional orientation (N2) and attentional distribution (P3) on food cues came earlier. The hunger hypothesis suggests that hunger can affect the processing of food cues. The results suggest that hunger can affect the processing of food cues. This effect was manifested in one study by an enhanced ability to deal with food stimuli to capture attention when participants were hungry (Piech et al., 2010). In another study, overweight or obese adults had an increased preference for high-energy, non-sweet food after moderate-intensity interval exercise (Alkahtani et al., 2014). Exercise could increase hunger and then activate the food reward system in overweight individuals, which would increase the attention to food cues. In addition, the longer attentional time and the lower attentional engagement index to the high-energy food cues were related to the larger amplitude of N2. Women who are overweight have been shown to demonstrate an attention bias toward high-energy food information and to invest more cognitive resources in high-energy food (Leng et al., 2021). In one study, high-energy food was a reward food for overweight groups, and attention bias to reward food information was the main reason for overweight (Newman et al., 2008). It can be suggested that there is an internal relationship between the behavioral and ERP results.

It should be emphasized that the RT analysis demonstrated neither significant group nor cue-congruence effects in our study findings. However, the neurophysiological results, particularly N2 and P3 components in terms of their latency and amplitude, demonstrated several significant differences related to group effects and cue-congruence effects. This may be attributed to the different measurement sensitivities of RT and ERPs. Compared to classical RT measurement, the ERP data seems to exhibit greater precision in capturing and evaluating attentional processes, which stems from its millisecond-level temporal resolution. Besides, the non-significant RT results can also be attributed to the low reliability and validity of the dot-probe task. The dot-probe paradigm, while widely established as a conventional method for measuring attentional bias, has faced increasing scrutiny in recent years due to concerns regarding its relatively low reliability and validity (Thigpen et al., 2018; Chapman et al., 2019; Xu et al., 2024). Even though the classical dot-probe tasks have been criticized, the integration of neurophysiological measures (i.e., EEG/ERPs), into the dot-probe paradigm, could still provide enhanced temporal resolution of attentional processes, offering mechanistic insights that extend beyond conventional behavioral data (i.e., RT), thereby significantly complementing and enriching the traditional experimental designs. It might be of interest for future studies using frequency-based EEG indicators together with the dot-probe task as well.

Limitations of the present study should be taken into consideration. As the experiment exclusively involved young female participants, the results could not be generalized to males or to other age groups. Moreover, it should be noted that potential menstrual cycle effects on food-related stimuli were not well controlled. Previous research (Frank et al., 2010; Yao et al., 2022) has demonstrated that menstrual cycle phases may significantly influence the attentional processing of food-related cues. For future research, it might be helpful to measure the menstrual cycle as a covariate. Besides, the hunger level, which may also affect participants’ attentional bias toward food cues, was not measured or well controlled in the experiment. We instructed participants to fast for two hours prior to the experiment, but individual hunger levels may still have varied among them. Future studies may take hunger levels into consideration (such as using blood sugar level to control it). Furthermore, a pre-test seems to be needed as well. Even though participants were randomly assigned to different groups, the attentional bias can still be different at the beginning.

## Conclusion and suggestions

The present study demonstrated that overweight female college students exhibited an attentional bias toward foods; however, such attentional bias could be mitigated by engaging in a 41-minute resistance exercise with moderate intensity. Following resistance exercise, overweight female university students exhibited accelerated early-stage conflict detection and expedited evaluative decision-making toward food cues, alongside enhanced inhibitory control specific to low-calorie food stimuli and attenuated automatic attentional capture by food-related stimuli. Critically, our findings demonstrate that resistance exercise reduces behavioral preferences for calorie-dense foods while promoting healthier dietary decision-making in this population. Further research is warranted on the effects of exercise on the attention bias toward food cues in terms of gender and short-term exercise. In terms of practical significance, this neurocognitive mechanism—potentially explaining the post-exercise shift toward healthier food choices—transcends established energy-centric frameworks, providing an empirical foundation for neuroscience-informed weight management interventions targeting overweight female adolescents.

## Ethical statement

The study was conducted according to the guidelines of the Declaration of Helsinki and approved by the Institutional Research Ethics Committee of the first author’s affiliated university (protocol code: 2022,018). Written informed consent has been obtained from the participants of the current experiment. The sponsors had no role in the design, execution, interpretation, or writing of the study.

## Data availability

Research data (both raw data and analyzed data) involved in the current study is available by contacting the first author or the corresponding author.

## Declaration of competing interest

The authors declare that they have no competing interests. The funders had no role in study design, data collection, data analysis, decision to publish, or preparation of the manuscript.
